# Novel induced *mlo *mutant alleles in combination with site-directed mutagenesis reveal functionally important domains in the heptahelical barley Mlo protein

**DOI:** 10.1186/1471-2229-10-31

**Published:** 2010-02-19

**Authors:** Anja Reinstädler, Judith Müller, Jerzy H Czembor, Pietro Piffanelli, Ralph Panstruga

**Affiliations:** 1 Max-Planck Institute for Plant Breeding Research, Department of Plant-Microbe Interactions, Carl-von-Linné-Weg 10, 50829 Köln, Germany; 2 Ulm University, Department for Molecular Genetics and Cell Biology, James-Franck-Ring N27, 89081 Ulm, Germany; 3 Plant Breeding and Acclimatization Institute (IHAR), Plant Breeding and Genetics Department, Radzikow, 05-870 Blonie, Poland; 4 The Sainsbury Laboratory, John Innes Centre, Colney, Norwich NR4 7UH, UK; 5 Parco Tecnologico Padano, Località Cascina Codazza, 26900 Lodi, Italy

## Abstract

**Background:**

Recessively inherited natural and induced mutations in the barley *Mlo *gene confer durable broad-spectrum resistance against the powdery mildew pathogen, *Blumeria graminis *f.sp. *hordei*. *Mlo *codes for a member of a plant-specific family of polytopic integral membrane proteins with unknown biochemical activity. Resistant barley *mlo *mutant alleles identify amino acid residues that are critical for Mlo function in the context of powdery mildew susceptibility.

**Results:**

We molecularly analyzed a novel set of induced barley *mlo *mutants and used site-directed mutagenesis in combination with transient gene expression to unravel novel amino acid residues of functional significance. We integrate these results with previous findings to map functionally important regions of the heptahelical Mlo protein. Our data reveal the second and third cytoplasmic loop as being particularly sensitive to functional impediment by mutational perturbation, suggesting that these regions are critical for the susceptibility-conferring activity of the Mlo protein. In contrast, only mutations in the second but not the third cytoplasmic loop appear to trigger the Endoplasmic Reticulum-localized quality control machinery that ensures the biogenesis of properly folded membrane proteins.

**Conclusion:**

Our findings identify functionally important regions of the polytopic barley Mlo protein and reveal the differential sensitivity of individual protein domains to cellular quality control.

## Background

The powdery mildew disease, caused by obligate biotrophic ascomycete fungi of the order Erysiphales, is a major impediment for cereal (e.g. wheat and barley) agriculture in temperate climates. In barley (*Hordeum vulgare*), polygenic resistance, dominantly inherited resistance (*R*) genes or recessively inherited mutants of the ***M****ildew resistance ****l****ocus ****o ***(*Mlo*) confer protection against the fungal disease [1]. While *R *genes usually provide isolate-specific resistance that is of little constancy under field conditions, *mlo *resistance is broad-spectrum and durable [2,3]. Mutants at the *Mlo *locus, which can be obtained by mutagenesis of any susceptible wild type line, were first described in the 1940s [2,4]. Besides a broad collection of induced *mlo *mutants [5-10] one natural *mlo *allele has been reported to date. This allele (*mlo*-11) originates from an Ethiopian landrace and represents the major source of *mlo *resistance introgressed into cultivated European spring barleys [2,11,12]. The *Mlo *gene encodes the founder of a family of plant-specific integral membrane proteins with heptahelical topology and yet unknown biochemical activity [13]. *Mlo *genes are organized in small- to medium-sized families per higher plant species [14-16]. Powdery mildew resistance conferred by loss-of-function alleles of the *Mlo *locus have for a long time thought to represent a unique feature of the monocot barley. Recently, however, *mlo *resistance was also discovered in the dicot species *Arabidopsis thaliana *and *Solanum lycopersicum *(tomato), either caused by induced mutant alleles or as a consequence of a naturally occurring deletion in the coding region, respectively [17,18].

Previous molecular analyses of induced barley *mlo *mutants in combination with site-directed mutagenesis and bioinformatic analysis based on multiple sequence alignments provided a first glimpse on the amino acids of the heptahelical barley Mlo protein that are decisive for its susceptibility-conferring function [19-23]. Amongst these residues are four extracellularly located cysteines that are thought to form two disulfide bridges as well as few amino acids located in various regions of the polytopic membrane protein. These studies also revealed that some mutation-induced single amino acid substitutions lead to aberrant Mlo variants that each serves as a substrate for an Endoplasmic Reticulum (ER)-associated quality control system. This cellular quality control system, also known as ERAD (Endoplasmic Reticulum-Associated Protein Degradation), is conserved among eukaryotes and recognizes malformed protein variants in the ER. These are then retranslocated to the cytoplasm, poly-ubiquitinated and subsequently degraded by the 26S proteasome [24,25]. Three distinct ERAD pathways can be defined on the basis of the different ubiquitin ligase complexes involved in substrate elimination: The ERAD-L pathway mediates removal of soluble (luminal) ER substrates whilst depending on the presence of either misfolded transmembrane or cytosolic domains, membrane-anchored substrates are removed by either the ERAD-M or ERAD-C pathway [26].

Here, we studied at the molecular level a set of hitherto uncharacterized natural and induced *mlo *mutant alleles to identify novel sites of functional relevance in the Mlo protein. We complemented this approach by site-directed mutagenesis based on alanine scan in a defined region of the Mlo protein. Our results reveal a crucial role for the second and third cytoplasmic loop in powdery mildew-associated Mlo function, while only lesions in the second cytoplasmic loop appear to be recognized by the cellular ERAD machinery.

## Results

### Identification of a putative novel natural *mlo *allele in an Ethiopian barley landrace accession

**Figure 1:**
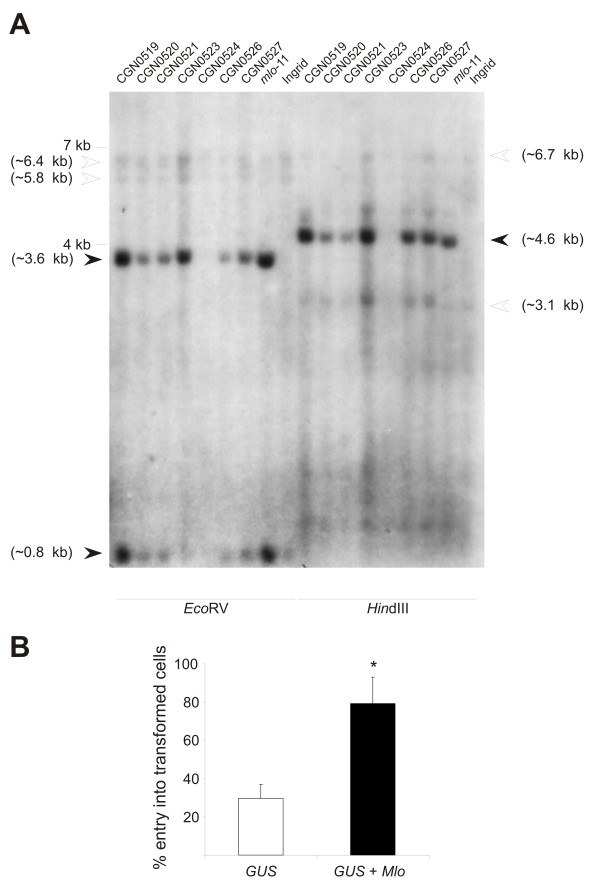
**Accession CGN0524 harbors a natural candidate *mlo *allele**. **A**. Southern blot analysis of natural *mlo *candidate accessions. Genomic DNA of indicated barley accessions was digested with either *Eco *RV or *Hin *dIII, blotted onto a nylon membrane and probed with a radiolabelled full-length *Mlo *cDNA fragment. White arrowheads indicate fragments of the wild-type *Mlo *copy, black arrowheads point to the prominent *mlo*-11-characteristic signals (see also [11]). Approximate sizes of these fragments were calculated based on the DNA sequence of the *Mlo *genomic locus [42] and the arrangement of the *mlo*-11-specific repeats [11]. **B**. Single cell complementation of accession CGN0524 by transient gene expression. Leaves of CGN0524 were either bombarded with a plasmid encoding the GUS reporter protein or co-bombarded with the GUS reporter plasmid and a plasmid harboring the wild type *Mlo *cDNA. Fungal entry rates in transformed (GUS-stained) cells were scored at 48 hours post inoculation. Results show the mean ± standard deviation of n = 3 experiments for expression of *GUS *alone and n = 5 experiments for expression of *GUS *+ *Mlo*. The asterisk indicates a statistically significant difference (p < 0.01) from the *GUS *control according to Student's t-test.

We previously identified the *mlo*-11 allele as a sporadic mutant in two-rowed awnless Ethiopian barley landraces that at the molecular level is characterized by the presence of a unique repeat structure [11]. To screen for further natural *mlo *alleles distinct from *mlo*-11, we examined additional powdery mildew resistant barley germplasm of different geographical origin by gel blot analysis for presence of the characteristic *mlo*-11 genomic tandem repeat structure. One source was Ethiopian barley landrace material from the Dutch Centre for Genetic Resources in Wageningen http://www.cgn.wur.nl/UK/. While seven of the eight tested accessions obtained from this site showed the hybridization pattern that is typical for the *mlo*-11 allele (a dominant band resulting from the repeated DNA region; Figure 1A), one accession (CGN0524) lacked this signal, suggesting that this line does not harbor the common natural *mlo*-11 allele. Microscopic evaluation revealed that the *Bgh *entry rate in this accession was ca. 30% (29.7% ± 7.5%; Figure 1B), considerably below the 50-80% penetration success that is typical for *Bgh *on susceptible wild type (*Mlo *genotype) barley lines, but clearly distinct from the near complete penetration resistance seen in *mlo *null alleles. We reasoned that this landrace either expresses a type of powdery mildew resistance that is different from *mlo*-conditioned resistance, or that it may contain a weak natural *mlo *allele with residual susceptibility-conferring activity. To differentiate between these two possibilities, we extracted RNA from accession CGN0524 and performed reverse transcription-polymerase chain reaction (RT-PCR) to amplify and sequence the *Mlo *cDNA sequence of this line. Compared to the reference sequence (GenBank accession number Z83834; [20]) we determined a G to C (G226C) exchange in the *Mlo *coding sequence resulting in a valine to leucine (V76L) substitution at the amino acid level. The predicted amino acid exchange is located in the second transmembrane domain of the heptahelical Mlo protein. The respective polymorphism is neither present in the *Mlo *sequence of a selection of European barley cultivars nor in the *Mlo *sequence of 40 analyzed barley *Hordeum spontaneum *accessions of broad haplotype diversity [11] and may therefore represent a novel natural *mlo *allele.

To further assess this hypothesis we used the previously described transient gene expression assay in single barley leaf epidermal cells [27] to analyze a potential complementation (i.e., restoration of wild type-like *Bgh *entry rates) of the partial penetration resistance in this accession upon overexpression of a wild type *Mlo *cDNA. This experiment resulted in the reinstatement of a *Bgh *entry rate that is typical for the complementation of *mlo *resistance in this assay (79.2% ± 13.9% host cell penetration; Figure 1B; compare with results in [28]). We thus conclude that the accession CGN0524 likely harbors a novel natural *mlo *allele that results in partial loss of *Mlo *function. Mutagen-induced weak *mlo *alleles with residual function have been previously reported (e.g. *mlo*-12 (F240L) and *mlo*28 (T222I); [23]) However, the *mlo*-typical recessive inheritance and allelism with known *mlo *mutants need to be formally proven by future test crosses of the accession with barley *Mlo *and *mlo *genotypes.

### Two landrace accessions from Yemen harbor the *mlo*-11 allele

Next we analyzed powdery mildew infection types of two barley landrace accessions (lines 5589 and 5590) collected in Yemen. Based on an inoculation survey with a broad range of powdery mildew isolates (J. Czembor, unpublished data), these two accessions, which originate from the Al Bayda' province of Yemen, were suspected to harbor a *mlo *allele. Indeed, first leaves of selfed progeny of these two lines (5589-1-1 and 5590-1-1) showed no visible signs of infection except for an occasional infection type 4 (compatible), resulting in few mildew colonies. These colonies were about half the size compared to colonies on the susceptible barley cultivar Manchurian. Such a reaction has been previously designated as 0/(4) and is characteristic of *mlo *resistance [2].

**Figure 2:**
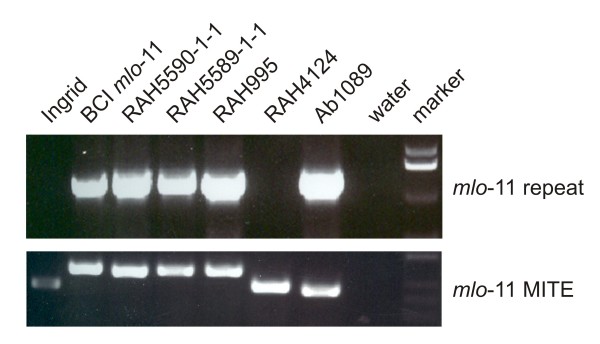
**Yemenite accessions 5589 and 5590 harbor the natural *mlo*-11 allele**. Genomic DNA of the indicated barley lines was used as a template for PCR amplification (40 cycles; extension 1 minute at 72°C) using either the oligonucleotide combination ADUP7A/Mlo6 (diagnostic for the presence of the *mlo*-11 repeat structure [11]) or Mlo6/Mlo10 (indicating presence/absence of a MITE associated with the 5'-terminal repeat in the majority of *mlo*-11 haplotypes [11,30]). PCR products were separated by agarose gel electrophoresis and visualized *via *ethidium bromide staining in combination with UV transluminescence. Cultivar Ingrid (*Mlo *genotype) and line back cross Ingrid (BCI) *mlo*-11 served as controls for wild type and *mlo*-11 genotypes, respectively. Note that lines RAH995 and Ab1089 were included as additional controls.

We first tested the possibility that the Yemenite accessions harbor the well-characterized *mlo*-11 allele. PCR analysis using genomic DNA and two specific primer pairs (one diagnostic for the presence of the *mlo*-11 repeat structure and the other indicating presence/absence of a Miniature inverted repeat transposable element (MITE) associated with the terminal repeat in the majority of *mlo*-11 genotypes) revealed that both 5589-1-1 and 5590-1-1 harbor the *mlo*-11-typical repeat units (Figure 2). We conclude that the two accessions collected in Yemen represent *mlo*-11 alleles, which were originally reported to come from Ethiopia [11].

### An accession from Turkey harbors the *mlo*-1 allele

Among 101 accessions of barley landraces collected before 1972 in Turkey that were screened for resistance to powdery mildew, four lines derived from landrace RAH4124 (also termed BGRC38917, collected in Konia/Anatolia; http://barley.ipk-gatersleben.de/genres/index.php?scp=barley&thm=matses&lev=acc&rec=BGRC%2038917) showed resistance to a set of 19 tested *Bgh *isolates. Resistance of these lines was thus suspected to be based on a mutation at the *Mlo *locus [29]. PCR analysis of genomic DNA from line RAH4124 indicated that this accession does not harbor the natural *mlo*-11 allele (Figure 2). We extracted total RNA from first leaves of the respective mutant plant and used it as template for RT-PCR-based amplification of the *Mlo *coding sequence. The amplified *Mlo *cDNA sequence was subjected to sequence analysis and found to harbor a T to A nucleotide substitution, predicted to result in a change of tryptophan 162 to arginine (W162R) at the protein level. This sequence polymorphism is identical to the *mlo*-1 mutant allele, which was originally induced in cultivar Haisa by X-ray mutagenesis [4]. The black seed coat of accession RAH4124 indicates, however, that this line is distinct from the European cultivars known to harbor the *mlo*-1 allele, which are typically characterized by light seed coats. To resolve whether line RAH4124 harbors an independent and possibly natural version of the *mlo*-1 mutant allele we performed molecular fingerprint analysis at the *Mlo *locus. We employed previously described simple sequence repeat (SSR) markers that are located few kb upstream of the *Mlo *gene and that were found suitable, owing to their highly polymorphic character, to display the haplotype diversity of cultivated and wild barleys [11,30]. PCR amplification and subsequent DNA sequence analysis revealed that the respective haplotype of accession RAH4124 is identical to that of the original *mlo*-1 mutant in cultivar Haisa as well to a *mlo*-1-containing backcross line (7 times backcrossed) in cultivar Ingrid (Table 1). Taken together, these results suggest that the original radiation-induced *mlo*-1 allele is present in accession RAH4124, likely because of its deliberate or accidental introgression into a barley line/variety from the Middle East that has a dark seed coat.

**Table 1:** Haplotype analysis at *Mlo*.

SSR/MITE marker ^1^	Barley line			
	**cv. Ingrid**	**Backcross Ingrid *mlo*-1**	***mlo*-1 (cv. Haisa)**	**RAH4124**

2259 (SSR)	(G)11 + CC **G **TT	(G)11 + CC **A **TT	(G)11 + CC **A **TT	(G)11 + CC **A **TT
4264 (SSR)	(G)13	(G)12	(G)12	(G)12
4801 (SSR)	(TA)7	(TA)7	(TA)7	(TA)7
7646 (MITE)	No polymorphism	No polymorphism.	No polymorphism.	No polymorphism

^1 ^for identity of markers and primers used, see Methods

### Molecular characterization of novel induced *mlo *alleles

Given the limited success to identify new natural *mlo *alleles we focused in the following on induced *mlo *alleles. We previously reported the molecular characterization of a range of *mlo *mutant alleles that were either induced by radiation or chemical mutagenesis [19,20,23]. To extend the collection of informative mutant sites we analyzed the *Mlo *coding sequence of a further range of induced *mlo *candidate alleles reported in the literature [2,31]. The respective mutant plants were previously found to exhibit the *mlo *characteristic 0/(4) infection phenotype, suggesting that they are affected at the *Mlo *locus. Moreover, for two of the mutant plants (*mlo*-2 and *mlo*-6) an allelic relationship with other *mlo *mutants has been shown [32].

We extracted total RNA from first leaves of the respective powdery mildew resistant mutant plants and used it as template for RT-PCR-based amplification of the *Mlo *coding sequence. Amplified *Mlo *cDNA sequences were subjected to sequence analysis. In most cases we identified single nucleotide polymorphisms that either lead to a single amino acid substitution or a premature stop codon (additional file 1). Two mutants (*mlo*-6 and *mlo*-44) revealed evidence for aberrant *Mlo *transcript splicing, owing either to the presence of multiple aberrant transcript versions (*mlo*-6; resulting in overlapping sequence traces) or the presence of one aberrant transcript type harboring the entire *Mlo *intron 3 (*mlo*-44).

In case of *mlo*-6, pooled cDNAs were cloned into vector pCR-BluntII-Topo and seven individual recombinant clones subjected to sequence analysis. This revealed a G to A mutation in the AG consensus 3' splice site of intron 4, resulting in four distinct versions of the *Mlo *cDNA, including a wild-type-like cDNA and three incorrectly spliced variants (data not shown). All anomalous splicing variants give rise to frame shifts and premature stop codons. Occurrence of a low level of correctly spliced cDNAs in the *mlo*-6 mutant indicates that the defective 3' consensus splice site is still being used, though seemingly with a lower efficiency. In case of *mlo*-44, a G to A mutation in the GT consensus 5' splice site sequence of intron 3 results in a complete lack of splicing of this intron. Consequently, the respective cDNA harbors the entire intron 3 sequence, leading to an aberrant coding sequence and a premature stop codon (data not shown).

Overall, we characterized 13 novel *mlo *alleles (*mlo*-2, *mlo*-6, *mlo*-34 to *mlo*-44), of which two harbor mutations in splice junctions (*mlo*-6 and -44), while four result in a premature stop codon (*mlo*-34, -36, -39 and -43) and seven give rise to single amino acid substitutions in various regions of the Mlo protein (*mlo*-2 (A349T), -35 (H231L), -37 (S71F), -38 (G318R), -40 (G264D), -41 (R209K), and -42 (S187L); additional file 1). Interestingly, one amino acid substitution (glycine 318 to arginine; G318R) was found twice, in lines SR59 (in the genetic background of cv. Bonus) and SR65 (in the background of cv. Kristina). Though it is a possibility that the same mutation was induced twice independently, it is more likely that a mix-up in seed materials resulted in duplication of the same mutant allele. Lack of DNA sequence polymorphisms in the *Mlo *cDNAs of cultivars Bonus and Kristina prevents discrimination of these two scenarios. Interestingly, the same amino acid residue (glycine 318) is affected in the previously described fully independent *mlo*-27 allele in the genetic background of cultivar Plena; however, in the latter mutant the glycine residue is replaced by glutamic acid (G318E; [23]; additional file 1).

### Site-directed mutagenesis of conserved amino acid residues in the second and third cytoplasmic loop of the Mlo protein

**Figure 3:**
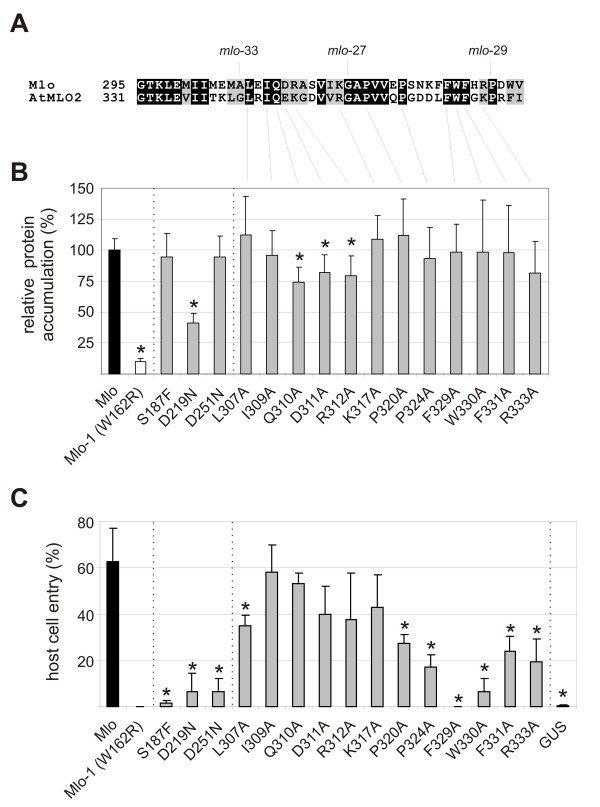
**Biological activity of Mlo variants with single amino acid substitutions in the second and third cytoplasmic loop**. **A**. Pairwise amino acid alignment of the third cytoplasmic loop of barley Mlo and AtMLO2. *mlo*-27 (G318E), *mlo*-29 (P334L) and *mlo*-33 (A306T) denote previously known barley *mlo *mutant sites. Highlighted in black are identical amino acid residues, in grey residues with similar biochemical properties. Lines indicate amino acids selected for site-directed mutagenesis. **B**. Relative protein accumulation of Mlo protein variants. Protein accumulation was determined using the dual luciferase assay using wild type Mlo as a positive control (set as 100%) and the Mlo-1 mutant variant (characterized by the W162R amino acid substitution) as a negative control. Values represent mean ± standard deviation based on 4 independent experiments with 2 technical replicates each. Asterisks indicate a statistically significant difference (p < 0.05) from Mlo wild type according to Student's t-test. **C**. Functional assay of Mlo protein variants. Leaf segments of the powdery mildew resistant barley line BCI *mlo*-3 were co-bombarded with the pUbi-GUS reporter plasmid and a plasmid encoding the indicated Mlo protein variant. Host cell entry was scored in GUS-stained cells attacked by powdery mildew sporelings. Expression of wild type *Mlo *served as a positive control, expression of *GUS *alone as a negative control. Values represent mean ± standard deviation based on at least 3 experiments with each ca. 100 investigated GUS-positive cells/construct. Asterisks indicate a statistically significant difference (p < 0.05) from the *GUS *control according to Student's t-test.

**Figure 4:**
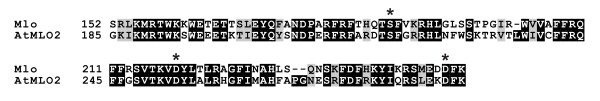
**Pairwise amino acid alignment of the second cytoplasmic loop of barley Mlo and AtMLO2**. Highlighted in black are identical amino acid residues, in grey residues with similar biochemical properties. Asterisks denote amino acids identified in powdery mildew resistant Arabidopsis *Atmlo2 *mutants [17].

Given the high prevalence of amino acid substitutions in the second and third cytoplasmic loop of the Mlo protein that result in non-functional Mlo protein variants we wondered whether conserved amino acid residues in these regions might be generally critical for the susceptibility-conferring activity. To address this question further we performed systematic site-directed mutagenesis in a defined portion of the third cytoplasmic loop. We selected a region located between the mutant sites of *mlo*-33 (A306T) and *mlo*-29 (P334L) and focused on amino acid residues in this part of the protein that are conserved with respect to their biochemical characteristics between the barley Mlo and *Arabidopsis thaliana *AtMLO2 co-orthologs [17] but distinct from the small aliphatic amino acids glycine, alanine and valine (Figure 3A). Nucleotide sequences in this stretch of the *Mlo *coding sequence were modified by PCR to encode alanine instead of the respective authentic amino acid (see Methods for details). This resulted in a set of twelve site-directed *mlo *mutants. Additionally, we generated barley *Mlo *cDNA analogs of three mutants originally discovered in *AtMLO2 *[17]. These comprise mutants *Atmlo2*-9, *Atmlo2*-10 and *Atmlo2*-11, which are each characterized by a single amino acid substitution in the *AtMLO2 *coding region and affect the second cytoplasmic loop of the AtMLO2 protein (S220F, D253N and D287N, respectively). We identified the corresponding residues in the barley Mlo protein by comparative amino acid sequence alignment (Figure 4) and created the respective mutant versions (S187F, D219N and D251N) by site-directed PCR mutagenesis (see Methods for details). Together with the twelve mutant variants of the third cytoplasmic loop, this resulted in a set of 15 novel single amino acid substitution variants for functional analysis of the Mlo protein.

We tested the fifteen mutant variants for their capacity to complement the *mlo *resistance phenotype in transient gene expression experiments [27] and additionally determined their *in planta *protein accumulation based on dual luciferase reporter assays, which were previously shown to faithfully reflect *in vivo *levels of Mlo mutant variants [25]. The latter analysis revealed that the accumulation of most mutant protein variants did not differ considerably from wild type Mlo (Figure 3B). Exceptions comprised mutant variants D219N, Q310A, D311A and R312A, which showed a statistically significant partial reduction in Mlo levels (ca. 40 to 80% relative accumulation compared to wild type Mlo). Whereas the tested variants largely retained wild-type like protein levels, most conferred a notable reduction in powdery mildew host cell entry. These included the three mutant variants S187F, D219N and D251N, which phenocopied the respective mutations in *AtMLO2 *with respect to powdery mildew resistance, as well as a range of mutant variants towards the end of the third cytoplasmic loop (Figure 3C). Of note in this respect is the F329A mutant that not even partially complemented the *mlo *resistance phenotype. Remarkably, lack of complementation was predominantly seen in mutant variants that showed unaltered (wild type-like) protein accumulation (compare Figures 3B and 3C). This excludes the possibility that lower Mlo protein levels account for the reduced functionality of these variants. Single amino acid substitution variants that showed impaired complementation efficiency, despite overexpression in the transient gene expression assay, denote amino acid residues that are crucial for Mlo function in the context of powdery mildew susceptibility.

## Discussion

**Figure 5:**
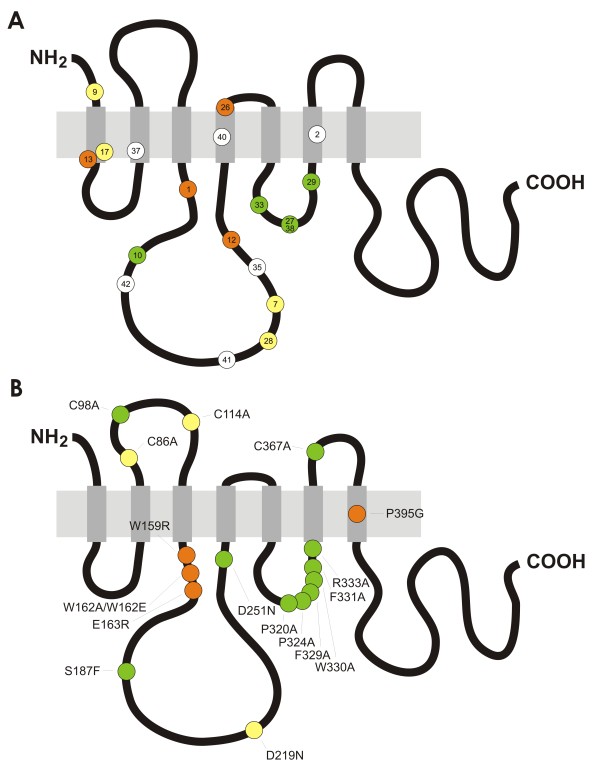
**A map of amino acid residues that are critical for Mlo function in the context of powdery mildew susceptibility**. Schematic representation of the barley Mlo protein and the relative position of loss-of-function single amino acid substitution mutations and a two amino acid deletion mutant (*mlo*-10). The light grey horizontal rectangle depicts the lipid bilayer, the grey vertical rectangles the seven transmembrane domains and the black bends the extracellular and cytoplasmic loops. NH_2 _and COOH denote the amino and carboxyl-terminus, respectively. **A**. Position of induced barley *mlo *mutations and relative protein accumulation of the respective protein variants. Colored dots designate barley *mlo *mutant alleles as defined in additional file 1. The color code indicates protein variants with wild type-like accumulation (green), intermediate accumulation (yellow), and severely affected accumulation (red), while white indicates lack of data about protein accumulation of this variant. **B**. Position of site-directed *mlo *mutants and relative protein accumulation of the respective protein variants. Colored dots designate the site-directed barley *mlo *mutants generated in this (Figure 3) and previous studies [21,22,25]. Only mutant variants with at least 50% reduction in fungal entry rate are shown. The color code indicates protein variants with wild type-like accumulation (green), intermediate accumulation (yellow), and severely affected accumulation (red).

In this study we molecularly characterized a variety of novel natural and induced *mlo *mutant candidates. This revealed one new natural candidate *mlo *allele as well as 13 chemically and radiation induced *mlo *alleles, comprising nine single amino acid substitutions that result in loss-of-Mlo function in the context of barley-powdery mildew interactions (additional file 1). Taken together, these protein sites can be integrated into a map of amino acid residues that are crucial for the powdery mildew susceptibility-conferring function of the Mlo protein (Figure 5). Intriguingly, most amino acids in this map cluster in the cytoplasmic regions of the protein, in particular in cytoplasmic loops 2 and 3. Although this picture is somewhat skewed by the fact that a range of single amino acid substitutions in the third cytoplasmic loop were selected in a targeted manner, it is noteworthy that all barley mutant alleles that result in stable Mlo protein variants (*mlo*-10 (ΔF182, T183), *mlo*-27 (G318E), *mlo*-29 (P334L) and *mlo*-33 (A306T)) are affected in amino acids that are located in cytoplasmic regions of the Mlo protein. Since information about the *in planta *protein accumulation of Mlo variants encoded by mutant alleles *mlo*-35 (H231L), *mlo*-41 (R209K) and *mlo*-42 (S187L) is lacking to date, this figure might be even higher. Moreover, the three single amino substitution variants that were engineered in the barley Mlo protein based on the data of powdery mildew resistant Arabidopsis *Atmlo2 *mutant alleles (S187F, D219N, D251N) also localize to a cytoplasmic loop. It is noteworthy that with the exception of site-directed mutants in cysteine residues [21] no barley *mlo *mutant was identified that codes for a mutation in an extracellularly localized amino acid. It thus appears that the cytoplasmic regions are of particular importance for Mlo function in the context of powdery mildew susceptibility. Cytoplasmic loop-loop interplay was previously suggested to be crucial for Mlo activity [21], and mutations in the cytoplasmic regions may interfere with these inter- or intramolecular domain interactions. Genetic analyses in Arabidopsis indeed suggest the possible formation of homo- or hetero-oligomeric Mlo complexes [17,33]. Alternatively, mutations in the cytoplasmic loops may abrogate interactions with other, yet unidentified, protein partners or impede the yet unknown biochemical activity of the Mlo protein. It remains to be shown whether mutant sites identified in the context of powdery mildew resistance also compromise the authentic Mlo function. Allele-specific correlation of the degree of powdery mildew resistance with the extent of early leaf senescence [34,35], a pleiotropic effect of *mlo *mutants that is thought to reflect a genuine Mlo activity [23], suggests that the protein domains that are crucial for both functions at least largely overlap.

**Figure 6:**
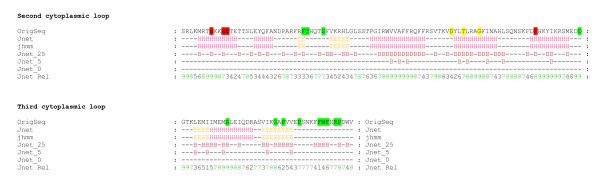
**Location of *mlo *mutant sites within the predicted consensus secondary structure of the Mlo second and third cytoplasmic loop**. Consensus secondary structures of the second and third cytoplasmic loop regions of monocot and dicot Mlo orthologs were calculated with the JPRED web tool (see Methods for details). Amino acids are given in the single letter code ("OrigSeq") and the final predicted secondary structure("Jnet") is indicated by a red H (α helix), a yellow E (β sheet) or dashes (random coil) below the sequence. The color code (highlighted residues) indicates amino acids that lead to non-functional (<50% entry rate compared to wild-type Mlo) mutant variants with wild type-like accumulation (green), intermediate accumulation (yellow), or severely affected accumulation (red). "hmm" indicates the Jnet hidden Markov model prediction; "Jnet_25", "Jnet_5" and "Jnet_0" signify the predicted relative solvent accessibility using 25%, 5% and 0% cut-off values. The prediction indicates whether a residue is buried ("B") or exposed ("-") at each of the three relative solvent accessibility cut-offs. "Jnet Rel" specifies the prediction accuracy for each position, ranging from 0 to 9 (the higher the better).

A set of characterized *mlo *mutants encode protein variants that show reduced Mlo accumulation (Figure 5; [25]). It was previously shown that these aberrant polypeptides are substrates of an ER-localized quality control system that monitors the integrity of membrane-localized proteins. This so-called ERAD machinery is conserved among eukaryotes and ensures that only properly folded proteins reach their final cellular destination. In the case of integral membrane proteins, the removal process of defective protein variants typically involves retrotranslocation from the ER membrane into the cytosol, polyubiquitination and subsequent degradation by the 26S proteasome. However, it is currently largely unknown which signatures classify malformed membrane proteins as clients of the ERAD machinery [24]. It has been suggested that cytoplasmic chaperones assist maintaining solubility of large cytoplasmic loops in ERAD-C substrates [24]. In this context it is remarkable that five out of seven tested Mlo mutant variants with amino acid substitutions in the large second cytoplasmic loop are ERAD substrates (encoded by *mlo*-1 (W162R), *mlo*-7 (G226D), *mlo*-12 (F240L), *mlo*-28 (T222I) and Mlo D219N), while the nine tested mutant variants of the small third cytoplasmic loop (encoded by *mlo*-27 (G318E), *mlo*-29 (P334L), *mlo*-33 (A306T), Mlo P320A, P324A, F329A, W330, F331A, R33A) are all stable *in planta*. We hypothesize that the larger second cytoplasmic loop is a major quality determinant of the Mlo protein, while mutations in the smaller third cytoplasmic loop appear to escape the ERAD machinery. This effect could be related to the size difference and/or the topology of the two cytoplasmic regions, which may result in a more prominent exposure of amino acid residues from the bulky second loop. Based on consensus secondary structure prediction of the second and third cytoplasmic loop (Figure 6) we noted that mutations resulting in unstable protein variants predominantly reside in regions that are predicted to adopt an α-helical structure. In contrast, mutations that escape the ERAD system are mostly located in regions that are predicted to adopt β-sheet or a random coil secondary structure. Thus, in addition to the differential recognition of the second and third cytoplasmic loop by their size, the secondary (and tertiary) structure of a given region might be the common denominator that determines detection by the ERAD quality control system.

Besides the second cytoplasmic loop, the transmembrane regions also seem to be sites that are particularly sensitive to mutational perturbations that result in reduced Mlo accumulation. This appears plausible since, in the case of integral membrane proteins, the delay or failure of mutant variants to co-translationally integrate into the ER lipid bilayer is known to lead to recognition by components of the ERAD-M pathway, relocation from the ER membrane and subsequent protein degradation. It is assumed that misfolded transmembrane domains expose hydrophobic amino acid side chains to the lipid bilayer, which are sensed by the transmembrane domain of the ERAD component Hrd1p, an ER-localized integral membrane protein with a C-terminal RING domain and E3 ubiquitin ligase function [36]. Notably, although they bare lesions in the cytoplasmic regions, the tested barley Mlo variants encoded by mutant alleles *mlo*-1 (W162R), *mlo*-12 (F240L) and *mlo*-13 (V30E) require Hrd1p for ERAD-based degradation in yeast [25].

In the context of the present study we also aimed to identify novel natural *mlo *alleles. Besides one presumptive *mlo *variant with a single amino acid polymorphism, we found the well-known *mlo*-11 allele in a set of additional powdery mildew resistant landraces and in two accessions from Yemen. The latter result challenges the proposed origin of the *mlo*-11 mutant in the Ethiopian highlands. Based on our new findings it remains at present unclear whether the *mlo*-11 mutant was introduced from Ethiopia to Yemen or *vice versa*. In principle, the *mlo*-11 allele may have originated even from another country of this geographical area. This example highlights the difficulties in tracing the roots and authentic population structure of wild plant communities in the context of anthropogenic perturbations such as agriculture, trading and, more recently, tourism. This problem is also exemplified by the *mlo*-1 mutation that was found to be introgressed into a presumed Turkish "landrace". Descriptive analyses of natural accessions should thus be accompanied by supporting molecular analyses whenever possible.

## Conclusions

We provide a comprehensive compilation of *mlo *mutants and their molecular defects that might be useful for plant breeding purposes and future research on Mlo protein function. Our data identifies the second and third cytoplasmic loop as functionally relevant sites of the polytopic barley Mlo protein. In contrast, only mutant variants with defects in the bulky second cytoplasmic loop serve as substrates for the cellular ERAD quality control system. The findings suggest the presence of molecular "hot spots" for the recognition of malformed protein variants by the ERAD machinery.

## Methods

### Plant and fungal material

Barley accessions CGN0519 1013, CGN0520 1014, CGN0521 1015, CGN0523 1016, CGN0524 1017, CGN0526 1018 and CGN0527 1019 were obtained from the Centre for Genetic Resources in Wageningen (The Netherlands; http://www.cgn.wur.nl/UK/).

Candidate barley *mlo *mutants SR34a, SR34b, SR34c, SR39a, SR39b, SR47, SR48, SR51a, SR51b, SR57, SR59, SR60, SR63, SR65, SR66, SR71, SR72 and SR73 were previously described [31] and kindly provided by the lab of U. Lundqvist (University of Lund, Sweden). Mutants *mlo*-2 and *mlo*-6 were made available by H. Jørgensen (Risø National Laboratory, Roskilde, Denmark), while lines backcross Ingrid (BCI) *mlo*-1, *mlo*-2, *mlo*-3 and *mlo*-6 were a gift from J. McKay (University of Uppsala, Sweden). Accessions 5589 and 5590 were collected in Yemen and kindly provided by J. Valkoun, J. Konopka and S. Ceccarelli (International Center for Agricultural Research in the Dry Areas - ICARDA, Aleppo, Syria), while accession RAH4124, collected in Turkey, was previously described [29]. *Blumeria graminis *f.sp. *hordei *isolate K1 was used for all experiments in this study. Fungal inoculum was regularly propagated on a susceptible barley line in a controlled growth chamber.

### Southern blot analysis

Genomic DNA of barley was digested with either *Eco *RV or *Hin *dIII and fragments separated by agarose gel electrophoresis. Upon transfer to a nylon membrane, the blot was probed with radiolabelled *Mlo *full-length cDNA in standard conditions.

### PCR-based detection of the *mlo*-11 repeat units

Presence of *mlo-11 *repeat units was determined by PCR using oligonucleotide primers ADUP7 (5' CTC AAG CTT GCC ACC ATG TCG GAC AAA AAA GGG G 3') and Mlo6 (5'-CAT CTA CTA CTA GCA TGT ACC-3'). This combination amplifies a ~1.2 kb *mlo*-11 repeat-specific fragment from genomic template DNA. Additionally, PCR reactions using oligonucleotide primers Mlo6 (for sequence, see above) and Mlo10 (5' GTC CTG CCA CCT AAG TAG CAG 3') were used. This combination amplifies a ~380 bp fragment from *Mlo *genotypes and a ~440 bp fragment from most *mlo*-11 genotypes [11,30].

### Haplotype analysis at *Mlo*

For haplotype analysis at *Mlo*, previously described microsatellite (2259, 4264 and 4801) and MITE markers (7646) were used. Oligonucleotide sequences for amplification of the markers from barley genomic DNA were reported in [11]. Amplicons were analyzed by direct DNA sequencing.

### RT-PCR and cDNA sequence analysis of *mlo *mutants

Total RNA was extracted from seven-day-old barley leaves using the Trizol reagent (Invitrogen, Carlsbad, CA) according to the instructions of the manufacturer. cDNA synthesis was carried out with Superscript II Reverse Transcriptase (Invitrogen, Carlsbad, CA) based on oligo-dT priming. *Mlo *cDNA was amplified by RT-PCR using oligonucleotides Mlo38 (5'- GCT TGC TCC GGG CAA GGA AGG-3', binding ca. 40 bp upstream of the start codon) and Mlo40 (5'-ATC ATC ACA TCC TAT GTT GGC-3', binding ca. 50 bp downstream of the stop codon) as forward and reverse primers, respectively. Sequences of *Mlo *cDNAs were determined using primers Mlo4 (5'-AAG GCG GAG CTC ATG CTG GTG GGC-3'), Mlo5 (5'-ACG CGT TAG AGC TAT GGA GAT GAC-3'), Mlo34 (5'-CGA TGG AGG ACG ACT TCA AGG-3') and Mlo1 (5' TGG TGG GGC TAG CTC TCC AG 3'). Sequencing was performed by the MPIZ DNA core facility on Applied Biosystems (Weiterstadt, Germany) Abi Prism 377, 3100 and 3730 sequencers using BigDye-terminator v3.1 chemistry. Premixed reagents were from Applied Biosystems. Oligonucleotides were purchased from Invitrogen (Carlsbad, CA). Partial *Mlo *cDNAs of mutant *mlo*-6 were cloned into vector pCR-BluntII-Topo using the Zero Blunt TOPO PCR cloning kit (Invitrogen, Carlsbad, CA) and DNA sequencing in this vector performed with the T7 primer.

### Site-directed mutagenesis of *Mlo *cDNA

Mutations were introduced into the *Mlo *wild-type coding sequence by PCR-based site-directed mutagenesis ("splicing by overlap extension"; [37]). Briefly, mutant sites were incorporated into a pair of complementary oligonucleotides. These primers were employed in combination with oligonucleotides representing the 5' and 3' termini of the barley *Mlo *gene for PCR amplification of the proximal and distal part of the *Mlo *cDNA (using cloned wild-type *Mlo *cDNA as a template). The two resulting PCR products were mixed, annealed and extended by few PCR cycles. The resulting full-length fragments were cloned into appropriate vectors and confirmed by DNA sequencing as described above.

### Transient gene expression in barley leaf epidermal cells

Ballistic transformation of detached barley leaves was carried out as previously described [21,38] using a Helios PDS1000 gene gun equipped with a Hepta adapter (BioRad, Hercules, CA). Bombarded specimens were inoculated with high densities of powdery mildew (*Blumeria graminis *f.sp. *hordei *isolate K1) conidiospores. Histochemical staining for β-glucoronidase (GUS) activity was performed at 48 hours post inoculation [38]. Epiphytic fungal structures were stained by Coomassie Brillant Blue (0.6% in ethanol). Leaf epidermal cells attacked by the appressorial germ tube of powdery mildew sporelings were microscopically evaluated for presence or absence of haustoria. Penetration success was calculated as the number of transformed cells that exhibit one or multiple haustoria in relation to the total number of transformed cells attacked by powdery mildew sporelings.

### Dual luciferase assay to monitor Mlo protein accumulation

Dual luciferase assays were performed as previously described [21,25]. We used plasmid K93 (derived of binary vector pAMPAT-MCS; GenBank accession number AY436765) that contains two separate expression cassettes: one consisting of a doubled cauliflower mosaic virus 35S promoter, an in frame fusion of *Mlo *and Renilla luciferase cDNAs and 35S terminator, the second comprising of 35S promoter, firefly luciferase and 35S terminator. Derivatives of this plasmid expressing *Mlo *variants as translational fusions with Renilla luciferase were generated by placement of suitable restriction fragments. *Arabidopsis thaliana *(ecotype Columbia 0, line At7) cells were propagated as reported in [39]. Protoplast preparations and transfections were carried out as described previously [40] using a modified enzyme solution containing 12 U/ml Cellulase Onuzoka R-10 (Merck, Darmstadt, Germany) and 1,5 U/ml Mazerozyme R-10 (Serva, Heidelberg, Germany). For dual luciferase reporter assays (Promega, Madison, USA) protoplast suspensions were diluted in 240 mM CaCl_2_, harvested by centrifugation (15.000 g), shock-frozen in liquid nitrogen and extracted in the lysis buffer supplied with the dual luciferase kit. Measurements of enzyme activities were performed according to the manufacturer's instructions. Renilla luciferase activity was set in relation to firefly luciferase activity and the ratio obtained with wild-type *Mlo *set as 100%.

### Consensus secondary structure prediction

The web-based tool JPRED http://www.compbio.dundee.ac.uk/www-jpred/ was used to calculate consensus secondary structures of the second and third loop of monocot and dicot Mlo orthologs (based on the sequences reported in [41]). Multiple sequence alignments of the relevant protein regions were generated by CLUSTALW http://www.ebi.ac.uk/Tools/clustalw2/index.html and the alignment uploaded for JPRED analysis in the "advanced" mode.

## Abbreviations

Bgh: Blumeria graminis, f.sp. *hordei*; ER: Endoplasmic Reticulum; ERAD: Endoplasmic Reticulum-Associated Protein Degradation; GUS: β-glucoronidase; RT-PCR: reverse transcription-polymerase chain reaction.

## Authors' contributions

AR conducted the molecular analysis of *mlo *alleles, generated plasmid constructs and performed the transient gene expression assays in barley; JM carried out the dual luciferase assays; JHC screened barley accessions; PP performed the Southern blot analysis shown in Figure 1; and RP designed the research and wrote the manuscript. All authors read and approved the final manuscript.

## Supplementary Material

Additional file 1 **Compilation of molecularly characterized *mlo *alleles**. This file provides an overview about the 36 *mlo *alleles characterized at the molecular level to date. It lists the mother variety, mutant ID, the mutagen, the mutational event at the DNA and protein level as well as references for each of the mutants. Click here for file
