# Supplementary material for: Novel induced mlo mutant alleles in combination with site-directed mutagenesis reveal functionally important domains in the heptahelical barley Mlo protein
Source: BMC Plant Biol. 2010 Feb 19;10:31. doi: 10.1186/1471-2229-10-31 (PMC2844067; doi:10.1186/1471-2229-10-31)
Supplement: Additional file 1 — Compilation of molecularly characterized mlo alleles. This file provides an overview about the 36 mlo alleles characterized at the molecular level to date. It lists the mother variety, mutant ID, the mutagen, the mutational event at the DNA and protein level as well as references for each of the mutants. [file 1471-2229-10-31-S1.DOC]

**Additional file 1. Compilation of molecularly characterized *mlo* alleles.**

| **Allele** | **Mother variety** | **Mutant ID** | **Mutagen ^a^** | **Mutational event at the cDNA level** | **Effect at the amino acid level** | **Reference mutant description** | **Reference molecular characterization** |
| --- | --- | --- | --- | --- | --- | --- | --- |
| *mlo*-1 | Haisa | M66 | X-rays | T^484^→A | W^162^→R | [4] | [20] |
| *mlo*-2 | Vollkorn | H3502 | X-rays | G^1045^→A | A^349^→T | [5] | this study |
| *mlo*-3 | Malteria Heda | M.C.20 | γ-rays | Δ^1188-1189^ | frame shift after F^395^ | [6] | [20] |
| *mlo*-4 | Foma | SR1 | X-rays | Δ^478-488^ | Frame shift after W^159^ | [7] | [20] |
| *mlo*-5 | Carlsberg II | R5678 | EMS ^a^ | G^3^→A | M^1^→I | [32] | [20] |
| *mlo*-6 | Carlsberg II | R6018 | EMS | G^985^→A ^c^ | n.a. ^b^ | [32] | this study |
| *mlo*-7 | Carlsberg II | R7085 | EMS | G^677^→A | G^226^→D | [32] | [20] |
| *mlo*-8 | Carlsberg II | R7372 | EMS | A^1^→G | M^1^→V | [32] | [20] |
| *mlo*-9 | Diamant | SZ5139b | EMS | C^28^→T | R^10^→W | [8] | [20] |
| *mlo*-10 | Foma | SR7 | γ-rays | Δ^543-548^ | Δ 2 amino acids (F^182^ and T^183^) | [7] | [20] |
| *mlo*-11 | n.a. | n.a. | n.a. | n.a. | n.a. | [12] | [11] |
| *mlo*-12 | Elgina | Do 4122 | NMU | C^720^→A | F^240^→L | [35] | [23] |
| *mlo*-13 | Plena | Do 2018 | EMS | T^89^→A | V^30^→E | [35] | [20] |
| *mlo*-16 | Alsa | Do 2376 | EMS | G^1917^A ^c^ | n.a. | [35] | [20] |
| *mlo*-17 | Plena | Do 2034 | EMS | C^92^→T | S^31^→F | [35] | [20] |
| *mlo*-26 | Plena | Do 2118 | EMS | T^809^→A | L^270^→H | [35] | [20] |
| *mlo*-27 | Plena | Do 2021 | EMS | G^953^→A | G^318^→E | [35] | [23] |
| *mlo*-28 | Nadja | Do 4228 | NaN_3_ | C^665^→T | T^222^→I | [35] | [23] |
| *mlo*-29 | Sultan 5 | n.a. | NaN_3_ | C^1001^T | P^334^→L | [23] | [23] |
| *mlo*-30 | Alsa | Do 2234, Do 2235 | EMS | A^2242^→T ^c^ | Δ 6 amino acids | [35] | [23] |
| *mlo*-31 | Ursula (six-rowed) | URS1 | NaN_3_ | ΔG^826^ | frame shift after G^276^ | [10] | [19] |
| *mlo*-32 | Prudentia (two-rowed) | PRU1 | NaN_3_ | G^103^→T | E^35^→stop | [10] | [19] |
| *mlo*-33 | Ursula (six-rowed) | URS2 | NaN_3_ | G^916^→A | A^306^→T | [10] | [19] |
| *mlo*-34 | Kristina | SR34a, SR34b, SR34c | EHOES | G^1269^→A | W^423^→stop | [31] | this study |
| *mlo*-35 | Kristina | SR39a, SR39b, SR47, SR48 | iso-PMS | A^692^→T | H^231^→L | [31] | this study |
| *mlo*-36 | Bonus | SR51a, SR51b | iso-PMS | G^1071^→A | W^357^→stop | [31] | this study |
| *mlo*-37 | Bonus | SR60 | NaN_3_ | C^212^→T | S^71^→F | [31] | this study |
| *mlo*-38 | Kristina | SR65 | iso-PMS | G^952^→A | G^318^→R | [31] | this study |
|  | Bonus | SR59 | NaN_3_ | G^952^→A | G^318^→R | [31] | this study |
| *mlo*-39 | Bonus | SR71 | iso-PMS | C^1051^→T | Q^351^→stop | [31] | this study |
| *mlo*-40 | Bonus | SR72 | NaN_3_ | G^791^→A | G^264^→D | [31] | this study |
| *mlo*-41 | Bonus | SR73 | NaN_3_ | G^626^→A | R^209^→K | [31] | this study |
| *mlo*-42 | Bonus | SR66 | X-rays | C^559^→T | S^187^→L | [31] | this study |
| *mlo*-43 | Bonus | SR63 | NaN_3_ | C^628^→T | Q^210^→stop | [31] | this study |
| *mlo*-44 | Bonus | SR57 | NaN_3_ | G678→A ^c^ | n.a. | [31] | this study |

^a^ EMS, ethyl methanesulfonate; NMU, nitrosomethylurea; EHOES, ethylhydroxy ethanesulfonate; iso-PMS, isopropyl methanesulphonate

^b^ n.a., not applicable

^c^ nucleotide designation according to genomic sequence (splice site mutation)
